# Interaction of land management and araucaria trees in the maintenance of landscape diversity in the highlands of southern Brazil

**DOI:** 10.1371/journal.pone.0206805

**Published:** 2018-11-21

**Authors:** Rafael Barbizan Sühs, Eduardo Luís Hettwer Giehl, Nivaldo Peroni

**Affiliations:** Department of Ecology and Zoology, Federal University of Santa Catarina (UFSC), Florianopolis, Santa Catarina, Brazil; Fred Hutchinson Cancer Research Center, UNITED STATES

## Abstract

In the southern Brazilian highlands, pre-Columbian societies created domesticated landscapes through the use and management of forests, including nurse *Araucaria angustifolia* trees, a common conifer in these regions. Nowadays, local smallholders still use traditional practices, such as burning, to promote vegetation for cattle grazing in highland grasslands. Even though burning is normally of small extent and low frequency, such management can slow down natural forest expansion and contribute to the maintenance of grasslands, by opposing the facilitative effect of nurse araucaria trees. To comprehend the interplay between human cultural management, species interactions and the environment, it is important to better understand how these relations affect diversity and composition. Our goal was to investigate how land management, biotic interactions and abiotic factors affect saplings species richness, abundance and composition. We hypothesized that (1) land management would decrease sapling richness and abundance and change sapling composition, (2) nurse araucaria trees would increase species richness and abundance and change sapling composition, and (3) the interactive effect between land management and nurse araucaria trees would shape sapling richness, abundance and composition. Data were collected in unmanaged and managed conditions, both beneath araucaria crowns and in nearby treeless areas. Our results indicate that abundance and species composition are affected by land management and araucaria crown influence. The highest values of sapling abundance were found beneath crowns in unmanaged areas. Species composition changed between all assessed combinations of land management and crown influence. Our study demonstrates the major roles of land management and facilitation in structuring communities, despite the effects of rock and grass cover. Moreover, our results clarify patterns and processes that may emerge in natural highland grasslands, such as the conversion of grasslands into forests and the loss of cultural landscapes when the main local management actions are excluded.

## Introduction

Plant communities are structured by local abiotic conditions, direct and indirect biotic interactions among plants, direct interaction with other organisms, stochastic processes, evolution, space and time [1]. Biotic interactions can promote changes in large-scale patterns of species distribution, affecting broader processes such as migration, speciation and extinctions [2]. The combination of negative interactions (*e*.*g*., competition, predation) and abiotic factors are the main components of most models of community structure and ecological theories [3,4]. However, positive interactions, such as mutualism and facilitation, are also important in shaping species composition, diversity and community dynamics (*e*.*g*., [5,6]). At the same time, past and present human activities help shaping large-scale patterns of species composition and community structure [7], having an important role in niche construction through domestication of animals and plants [8]. Domestication is here defined as co-evolutionary mutualisms that develop through active niche construction by both humans and plants or animals [8]. Domestication of animals and plants, and soil modification may result in domesticated landscapes with high levels of productivity and greater diversity of niches than landscapes without human actions [8,9].

Facilitation is a major mechanism in diversity maintenance [3,10,11], therefore contributing to niche construction. Facilitator species alter the environment and can mitigate potentially limiting stressors, thereby creating suitable habitats for other species [4], whereas the absence of a facilitator species in regions susceptible to climate change may slow down migratory processes, leading to species extinctions [12]. Facilitation tends to increase with physical severity of the environment (*i*.*e*., abiotic stress or high herbivore pressure) [5,11]. In severe habitats, such as elevated regions (highlands), facilitation may be more important than negative interactions [13,14]. Facilitation by shrubs and trees usually ameliorates environmental conditions because these plants provide shade and increase soil humidity, oxygenation and nutrient availability beneath their crowns [15,16]. Abiotic structures like rocks can also be beneficial to plant species, for example, by changing microclimatic conditions or protecting against fire [17–19]. These local scale changes induced by habitat amelioration may determine broader patterns and processes, overcoming regional-scale factors, such as climate [20].

Although there is interplay between human cultural management, species interactions and the environment, how these factors affect diversity, composition and species distribution is still poorly understood [7,21]. To understand current landscape dynamics, it is crucial to recognize past processes that have shaped terrestrial and aquatic ecosystems around the world for millennia [7]. In South America, domesticated landscapes created by pre-Columbian societies can be found in tropical [9,22] and subtropical forests and grasslands [21]. In southern Brazil, hunter-gatherer human societies arrived around 12 kyr BP and several societies successively occupied the highlands, within Araucaria forest domains [23]. These societies used several forest resources, such as wood, fruits and seeds, and probably also contributed to seed dispersal [23]. The use and management of forest resources, intensified at around 2.5 kyr BP with the arrival of different traditions of sedentary and agricultural societies (Tupi and Macro-Jê) [23,24]. Following the arrival of European settlers around 500 years BP, wars and diseases decimated most traditional populations, settlements, cultures and landscapes [21,23,24].

Araucaria forests (mixed rain forests) occur mostly throughout the southern Brazilian plateau [25], at altitudes varying from 500 m to 1800 m a.s.l. [26]. In these forests, the dioecious conifer *Araucaria angustifolia* (hereafter “araucaria”) is abundant and dominates the tree stratum [27]. At high altitudes (above 900 m a.s.l.), forests frequently form mosaics associated with shrubs and grasslands, the latter considered relicts of a past climate and likely maintained by fire and/or human activities [27]. The presence of araucaria within subtropical forests has raised questions of a possible past expansion of these forests into grasslands [27]. Indeed, around 4 kyr BP the regional climate became wetter, leading to a slow expansion of Araucaria forests into grasslands [28–30]. Around 1.5 kyr BP, however, the expansion was much faster and fire became more frequent than before, while the climate remained the same [30]. This sudden expansion matches the arrival of pre-Columbian societies in the region [21,31]. Previous studies have shown that as a consequence, domesticated landscapes were created through forest use and management [8], including the management of araucaria trees, whose seeds constituted part of the diet of these peoples [21,31]. Nowadays, local smallholders regularly use fire to promote grassland for cattle grazing [32]. Yet, such management may slow down natural forest expansion and contribute to the maintenance of grasslands [33,34], which are threatened by a series of factors, including forest expansion [34]. Additionally, grasses can hinder woody plant establishment through light and resource competition [35,36]. Araucaria trees, on the other hand, promote woody plant establishment because adult trees of this species act as perches for seed dispersers [37,38] and ameliorate conditions beneath their crowns by attenuating high temperatures in summer and increasing soil nutrient availability [16].

Conifers play an important role in structuring communities in high-altitude ecosystems across the globe (*e*.*g*., [18,20,39]). In Brazil, Araucaria forests and associated highland grasslands are highly threatened. Araucaria forests currently occupy around 12% of their original area [21,40] and *Araucaria angustifolia* is at risk of extinction [41]. In addition, Brazilian grasslands are often neglected by conservation policies and are threatened by several factors, including invasion by exotic species [42], mismanagement practices [34] and climate change [43]. Subtropical highland grasslands deserve attention not only because of these threats but also due to high levels of endemism, species richness and genetic diversity [44].

We carried out a field study in an upper-montane Araucaria forest-grassland mosaic to investigate how land management, biotic interactions and abiotic factors affect saplings species richness, abundance and composition. By testing the following hypotheses, we tried to understand how grasslands are maintained by management practices, how araucaria trees promote forest expansion, and the role of biotic interactions and abiotic factors involved in these processes. We hypothesized that (1) land management would decrease sapling richness and abundance and change sapling composition, (2) nurse araucaria trees would increase species richness and abundance and change sapling composition, and (3) the interactive effect between land management and nurse araucaria trees would shape sapling species richness, abundance and composition. We expect negative effects of land management because burning and grazing can kill or damage seedlings and saplings [45]. Conversely, nurse araucaria trees shade grasses, reducing their competitive ability, and favor recolonization by woody species because of a perch effect and facilitation [16,37]. Finally, we expect that the effect of management would override the nurse effect of araucaria trees.

## Materials and methods

### Study site

The study was conducted in the highlands of southern Brazil, in São Joaquim National Park (Lat 28.19°S, Lon 49.53°W), which is located within the municipalities of Bom Jardim da Serra, Urubici, Lauro Müller, Grão Pará and Orleans, in Brazil. This protected area has 49,300 hectares, of which 13,000 have been effectively protected (*i*.*e*. acquired by the Brazilian government) since 2006. The main vegetation types in the protected area include high-altitude grasslands, mixed rainforest (Araucaria forest) and tropical rainforest (Atlantic rainforest) [25]. The climate between 1961 and 2016, recorded by the nearest weather station (ca. 30 km), was characterized by an annual mean rainfall of 1,626.3 mm.yr^-1^, equally distributed throughout the year, and an annual mean temperature of 13.3°C. The average minimum temperature for the coldest month (July) was 6.0°C and the average maximum temperature for the hottest month (January) was 22.9°C. The minimum absolute temperature recorded was -9.0°C and the maximum absolute temperature was 31.4°C. During winter, frosts are common and it occasionally snows. Climate data compiled from [46]. In the region, especially in high-altitude grasslands, usual management actions consist of using fire every two or three years and removing shrubs to promote grassland vegetation for extensive livestock farming.

### Sampling methods and data collection

Data was collected in two locations, in 2015 and 2016. One location is situated inside the national park (Lat 28.142°S, Lon 49.631°W) and has not been managed (with fire, cattle and shrub removal) since 2008 (hereafter: “unmanaged”). The second location is a private property situated outside the protected area (Lat 28.142°S, Lon 49.644°W) and is currently managed (hereafter “managed”). The authorization for developing this study in the protected area and private property was approved by the Brazilian government (SISBio project code 48898–1) and the land owner, respectively. These locations are geographically close to each other (ca. 800 m away) and have the same climate (humid subtropical), soil type, elevation (ca. 1,450 m a.s.l.) and vegetation (mosaic of grasslands and shrubs among araucaria trees), and the terrain has a similar slope and aspect. There are no major environmental differences among the locations (more details in S1 Fig). In these locations, *Baccharis uncinella* DC. is the most common shrub, while *Scleria sellowiana* Kunth. and *Andropogon lateralis* Nees are the most common grasses. The locations were equally managed (fire and cattle grazing) until 2008, when the unmanaged location became protected. In the managed location, the last fire occurred in 2014 and there is an average of 0.15 cattle per hectare. These locations represent well the regional vegetation type with traditional land use history.

Isolated adult (>20 cm DBH) araucaria trees were randomly selected across the two locations, totaling 70 individuals in the unmanaged and 30 individuals in the managed location. This sampling difference is because the managed location has less trees than the unmanaged location. However, to keep the same variation in the environment, we tried to keep the same distance, for both locations, from the first selected tree to the last selected tree. The selection criteria were that trees should be mature and the next tree should be at least 10 meters apart from the previous selected tree [37]. Trees were sampled in one direction (from north to south) in both locations, within the same mosaic type. The areas covered by the surveys correspond to approximately 8 ha in the unmanaged location and five hectares in the managed location. Vegetation and environmental data were collected beneath crowns and near (hereafter “treeless areas”) araucaria trees, resulting in paired samples. Samples in treeless areas were randomly placed (drawn from cardinal points) at two meters away from the limits of the crown of the sampled araucaria tree and had no influence of any other tree species crown. Because we sampled the whole area beneath the crown of araucaria trees, the sample area in treeless areas was adjusted to match the area sampled beneath the crown. Thus, we also recorded the area of each sample, and the height and crown diameter of each araucaria tree with a digital laser distance meter. We recorded the identity and number of individuals of each sapling that was 30–200 cm tall beneath the crown of each selected araucaria tree and in the paired treeless areas. Species were identified in the field using scientific literature. Species nomenclature and classification follow the Brazilian Flora checklist [47].

To assess the role of covariates that can also affect woody seedling establishment, four squares (0.5 × 0.5 m) subdivided in four quadrats (0.25 × 0.25 m) were placed towards the geographical cardinal points, both beneath crowns and in the sampled treeless areas. We treated as covariates the cover of rock, shrub (*Baccharis uncinella*) and grasses. In addition, the average height of grasses was measured in each quadrat with a wooden ruler (1 cm precision). Although other graminoids were present and measured, they are hereafter referred to as only “grasses.” Grass volume was estimated as the product of grass cover and average grass height. Data collected from quadrants was averaged for each of the four squares and then averaged for each sample.

### Data analysis

We built mixed models (GLMM) using either species richness or abundance of saplings, as response variables and generalized linear model for multivariate responses (GLM_mv_) using community composition as response variables. For both types of models, land management, araucaria crown influence, grass volume, rock and shrub cover were treated as fixed effects terms. Models were built considering the hierarchical arrangement of variables, such as land management and araucaria crown influence affecting shrub cover and grass volume. Thus, we considered land management and crown influence as top variables, and therefore the effects of grass volume, rock and shrub cover were nested within the interaction between the two top variables. Based on this assumption, along with biological relevance and the hypotheses to be tested [48], a total of eight candidate models plus an intercept-only model (null) were built. To account for sampling pairing (beneath crowns and treeless areas), we treated pairs of samples (blocks) as random effects terms for GLMMs. We specified contrasts of factor levels for each model and adjusted P-values for multiple comparisons via the Holm-Bonferroni method.

For multivariate data (species composition), we also checked which species were significantly affected by land management, araucaria crown influence, grass volume and rock and shrub cover with univariate tests (using GLMs). To control for the paired sampling design, a permutation matrix was generated, where pairs of samples (blocks) were fixed, but blocks and factor levels within blocks (beneath crowns / treeless areas) were randomized. P-values were calculated based on the 10,000 matrices via PIT-trap resampling (adjusted for multiple testing) calculated using a stepdown resampling algorithm [49]. To visually check for changes in the community composition of sites differing in land management and crown influence, we explored the data with non-metric multidimensional scaling (nMDS). Before running the models, outliers were removed (3 samples) through graphical exploratory data analysis.

For all models, we chose the negative binomial distribution because it visually fit the residuals better compared to Poisson distribution [48]. To account for variations in sample size, we used sampling area as an offset in the models. Sampling area was log-transformed to match the scale of the modeled response (link function is log for negative binomial family). Model selection was based on the Akaike information criterion (AIC) and validated by a graphical analysis of residuals [48]. Finally, we assessed model performance through marginal and conditional R^2^ for GLMM (following [50]) and pseudo *R^2^* for GLM_mv_ (following [51]). We checked for spatial autocorrelation through correlograms that test for autocorrelation in the residuals and there was no clear pattern of decreasing autocorrelation with distance. All analyses were run in the R environment [52] using the “vegan” [53] package for producing the ordinations, “glmmADMB” [54] for GLMMs and “mvabund” [49] for GLM_mv_. An example of the mixed model built is the following:

Richness ~ (LM×CI)+LM/CI/Rock+LM/CI/Shrub+LM/CI/Grass+offset(log(Area))+(1|Block)

Where LM = land management (managed / unmanaged), CI = araucaria crown influence (beneath crowns / treeless areas), Rock = rock cover, Shrub = shrub cover, Grass = grass volume, area = sampling area, Block = blocks.

## Results

### Sapling richness

A total of 19 woody native species were found (no alien species were found) (S1 Table). The model best fitting differences in species richness contained land management, crown influence and grass volume as predictors (S2 Table). This model had the lowest AIC from the set of candidate models (Table 1) and residuals were visually adequate. Fixed effects accounted for 33% of the differences in species richness (marginal R^2^), reaching 62% when considering both fixed and random effects (conditional R^2^).

**Table 1:** Set of produced models for evaluating sapling species richness, abundance and composition in an upper-montane Araucaria forest, southern Brazil.

Model	Model ID	Int	CI × LM	CI × LM × Rock	CI × LM × Shrub	CI × LM × Grass	df	logLik	AIC	Delta AIC	AIC Weights
**Sapling species richness**	ric.7	-3.25	+			+	10	-351.6	723.2	0.00	0.588
	ric.2	-3.65	+	+		+	14	-348.8	725.6	2.42	0.175
	ric.8	-3.04	+				6	-356.9	725.7	2.57	0.162
	ric.5	-3.26	+	+			10	-354.6	729.1	5.98	0.030
	ric.3	-3.26	+		+	+	14	-350.7	729.5	6.32	0.025
	ric.1	-3.71	+	+	+	+	18	-347.6	731.1	7.98	0.011
	ric.6	-3.05	+		+		10	-355.9	731.9	8.73	0.007
	ric.4	-3.32	+	+	+		14	-353.4	734.9	11.69	0.002
	null	0.80					3	-394.7	795.4	72.2	0.000
	Variable Weight		1.00	0.22	0.04	0.80					
**Sapling abundance**	abu.2	-3.43	+	+		+	14	-506.3	1040.5	0.00	0.594
	abu.1	-3.54	+	+	+	+	18	-502.9	1041.8	1.3	0.310
	abu.5	-3.00	+	+			10	-513.0	1046.0	5.51	0.038
	abu.7	-2.58	+			+	10	-513.4	1046.8	6.28	0.026
	abu.4	-3.07	+	+	+		14	-509.6	1047.1	6.58	0.022
	abu.3	-2.58	+		+	+	14	-510.6	1049.3	8.73	0.008
	abu.8	-2.44	+				6	-520.2	1052.4	11.83	0.002
	abu.6	-2.42	+		+		10	-516.9	1053.7	13.17	0.001
	null	1.70					3	-565.6	1137.2	96.65	0.000
	Variable Weight		1.00	0.96	0.34	0.94					
**Sapling Composition**	com.8		+				5	-1676.5	3363.1	0.00	0.999
	com.7		+			+	9	-1692.9	3403.7	40.67	< .001
	com.6		+		+		9	-1710.4	3438.7	75.66	< .001
	com.5		+	+			9	-1716.3	3450.6	87.49	< .001
	com.2		+	+		+	13	-1726.7	3479.4	116.28	< .001
	com.3		+		+	+	13	-1736.1	3498.3	135.22	< .001
	com.4		+	+	+		13	-1750.3	3526.6	163.57	< .001
	com.1		+	+	+	+	17	-1766.5	3567.0	203.90	< .001
	null						2	-1798.3	3600.6	237.55	< .001
	Variable Weight		1.00	< .001	< .001	< .001					

Int = Intercept; CI = Araucaria crown influence; LM = land management; Rock = rock cover; Shrub = shrub cover; Grass = grass volume; df = degrees of freedom; logLik = log likelihood; “+” sign stands for inclusion in the referred model. For species composition models, logLik and AIC values are expressed as the sum of these parameters from the species univariate GLM. Interaction between predictors is represented by “×”.

**Fig 1:**
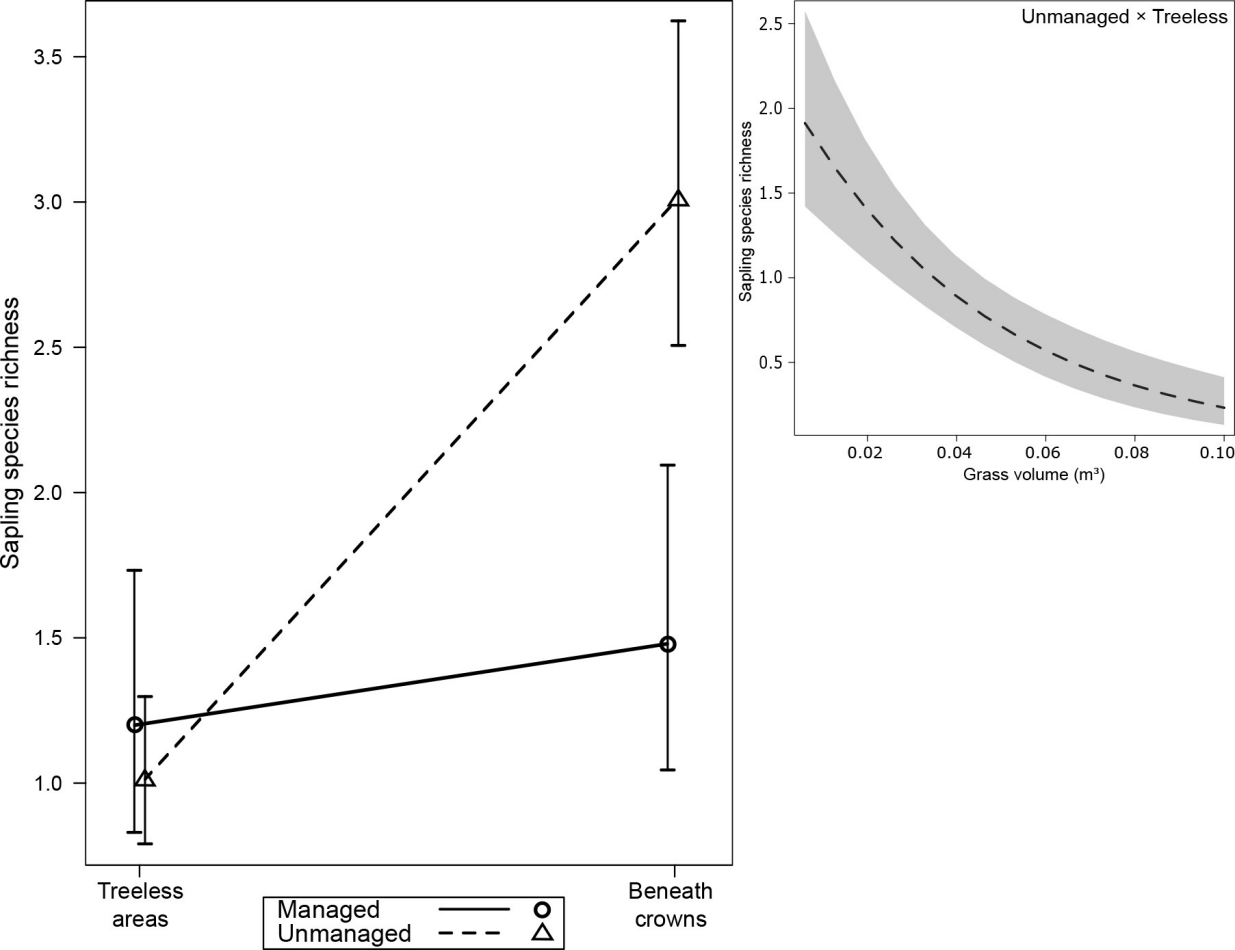
Species richness of saplings (species per m^2^) in relation to land management, araucaria crown influence and grass volume in an upper-montane Araucaria forest, southern Brazil. Shaded areas represent 95% confidence intervals. Effects of land management and araucaria crown influence on richness were plotted without the effects of grass volume. Effect of grass volume on richness was plotted from a GLM.

Beneath crowns, sapling species richness was three times higher in unmanaged than in managed areas (Estimate = -1.09, Std. Error = 0.31, z = -3.54, P_(adj.):_ = 0.002; Fig 1 and Table 2). Grass volume correlated negatively with species richness in treeless areas (Estimate = -19.91, Std. Error = 7.79, z = -2.55, P = 0.0107), but was uncorrelated with species richness beneath crowns (S2 Table).

**Table 2:** Comparisons among groups of land management and araucaria crown influence of selected models for evaluating saplings species richness and abundance in an upper-montane Araucaria forest, southern Brazil. These results are represented in Figs 1 and 2.

Model	Model ID	Coefficient comparisons			Estimate	Std.Error	z	P_(adj.)_
Sapling species richness	ric.7	Unman × Crowns	vs.	Unman × Treeless	-0.458	0.324	-1.41	0.628
		Unman × Crowns	vs.	Man × Treeless	0.973	0.524	1.86	0.316
		Unman × Crowns	vs.	Man × Crowns	-1.420	0.387	-3.67	**0.001**
		Unman × Treeless	vs.	Man × Crowns	-0.973	0.524	-1.86	0.316
		Unman × Treeless	vs.	Man × Treeless	-0.447	0.456	-0.98	0.654
		Man × Crowns	vs.	Man × Treeless	0.515	0.412	1.25	0.634
Sapling abundance	abu.2	Unman × Crowns	vs.	Unman × Treeless	-1.122	0.382	-2.94	**0.013**
		Unman × Crowns	vs.	Man × Treeless	2.085	0.616	3.39	**0.004**
		Unman × Crowns	vs.	Man × Crowns	-2.393	0.497	-4.81	**< .0001**
		Unman × Treeless	vs.	Man × Crowns	-2.085	0.616	-3.39	**0.004**
		Unman × Treeless	vs.	Man × Treeless	-0.308	0.551	-0.56	1.000
		Man × Crowns	vs.	Man × Treeless	0.962	0.483	1.99	0.139

Significant P-values after adjustment for multiple comparisons are in bold. Covariates were included in these models but are presented in a separate table. Unman = unmanaged sites; Man = managed sites; Crowns = beneath araucaria crowns; Treeless = treeless areas. Interaction between predictors is represented by “×”.

### Sapling abundance

A total of 1,300 sapling individuals were sampled (S1 Table). The model best fitting differences in abundance of saplings included land management, araucaria crown influence, grass volume and rock cover as predictors (S2 Table). This model had the lowest AIC from the set of candidate models (Table 1) and residuals were visually adequate. Fixed effects accounted for 50% of the differences in abundance (marginal R^2^), reaching 89% when considering both fixed and random effects (conditional R^2^).

**Fig 2:**
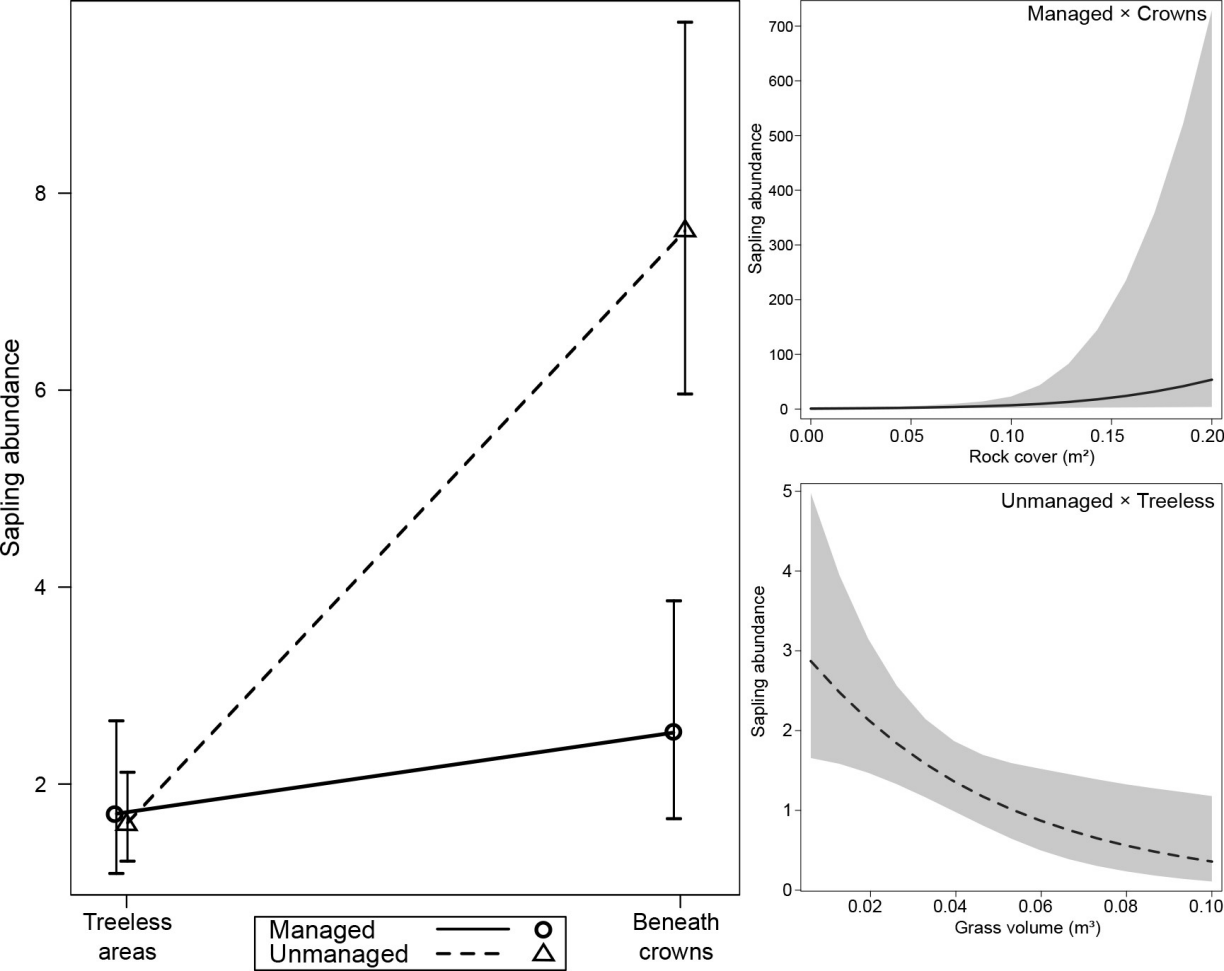
Abundance of saplings (individuals per m^2^) in relation to land management, araucaria crown influence and covariates (grass volume and rock cover) in an upper-montane Araucaria forest, southern Brazil. Shaded areas represent 95% confidence intervals. Effects of land management and araucaria crown influence on abundance were plotted without the effects of covariates (rock cover and grass volume). Effects of rock cover and grass volume on abundance were plotted from a GLM.

Sapling abundance was 12 times higher in the unmanaged-beneath crowns condition than in the managed-beneath crowns condition (Estimate = -2.39, Std. Error = 0.49, z = -4.81, P_(adj.)_ < 0.001), 5 times higher than managed-treeless areas (Estimate = 2.08, Std. Error = 0.61, z = 3.39, P_(adj.)_ = 0.004), and 3 times higher than unmanaged-treeless areas (Estimate = -1.12, Std. Error = 0.38, z = -2.94, P_(adj.)_ = 0.013). In unmanaged-treeless areas, sapling abundance was 3.5 times higher than in the managed-beneath crowns condition (Estimate = -2.08, Std. Error = 0.61, z = -3.39, P_(adj.)_ = 0.004; Fig 2 and Table 2). In unmanaged conditions, grass volume correlated negatively with sapling abundance in treeless areas (Estimate = -19.02, Std. Error = 8.83, z = -2.15, P = 0.031), but not beneath crowns. In managed conditions, rock cover correlated positively with sapling abundance beneath crowns (Estimate = 18.07, Std. Error = 5.24, z = 3.45, P<0.001), but was uncorrelated with abundance in treeless areas. No association of rock and shrub cover with abundance was found in unmanaged areas (S2 Table).

### Sapling composition

Land management and araucaria crown influence were the main predictors of sapling composition. The model containing these two variables had the lowest AIC from the set of candidate models (Table 1) and residuals were visually adequate. The model explained 15% of the variation in species composition (pseudo R^2^ for GLM_mv_). Species composition beneath crowns differed from treeless areas in both unmanaged (likelihood ratio test, LR = 214.20, P_adj._ < 0.001) and managed conditions (LR = 31.40, P_adj._ = 0.001). In treeless areas, species composition differed in both unmanaged and managed conditions (LR = 21.03, P_adj._ = 0.04). Similarly, beneath crowns, species composition differed between unmanaged and managed conditions (LR = 73.70, P_adj._ < 0.001). Finally, species composition was distinct between unmanaged-treeless areas and managed-beneath crowns conditions (LR = 31.40, P_adj._ = 0.001) and between managed-beneath crowns and managed-treeless areas conditions (LR = 36.69, P_adj._ < 0.001) (Table 3).

**Table 3:** Pairwise comparisons of species composition under different land management and araucaria crown influence in an upper-montane Araucaria forest, southern Brazil.

Model	Model ID	Coefficient comparisons			LR		P_(adj.)_	
Sapling composition	com.8	Unman × Crowns	vs.	Unman × Treeless	214.2		**0.0006**	
		Unman × Crowns	vs.	Man × Treeless	31.4		**0.001**	
		Unman × Crowns	vs.	Man × Crowns	73.7		**0.0006**	
		Unman × Treeless	vs.	Man × Crowns	31.4		**0.001**	
		Unman × Treeless	vs.	Man × Treeless	21.0		**0.039**	
		Man × Crowns	vs.	Man × Treeless	36.7		**0.0006**	

LR = likelihood ratio test. Unman = unmanaged sites; Man = managed sites; Crowns = beneath araucaria crowns; Treeless = Treeless areas. Bold values indicate significant P-values after Holm-Bonferroni correction for multiple testing. Interaction between predictors is represented by “×”.

**Fig 3:**
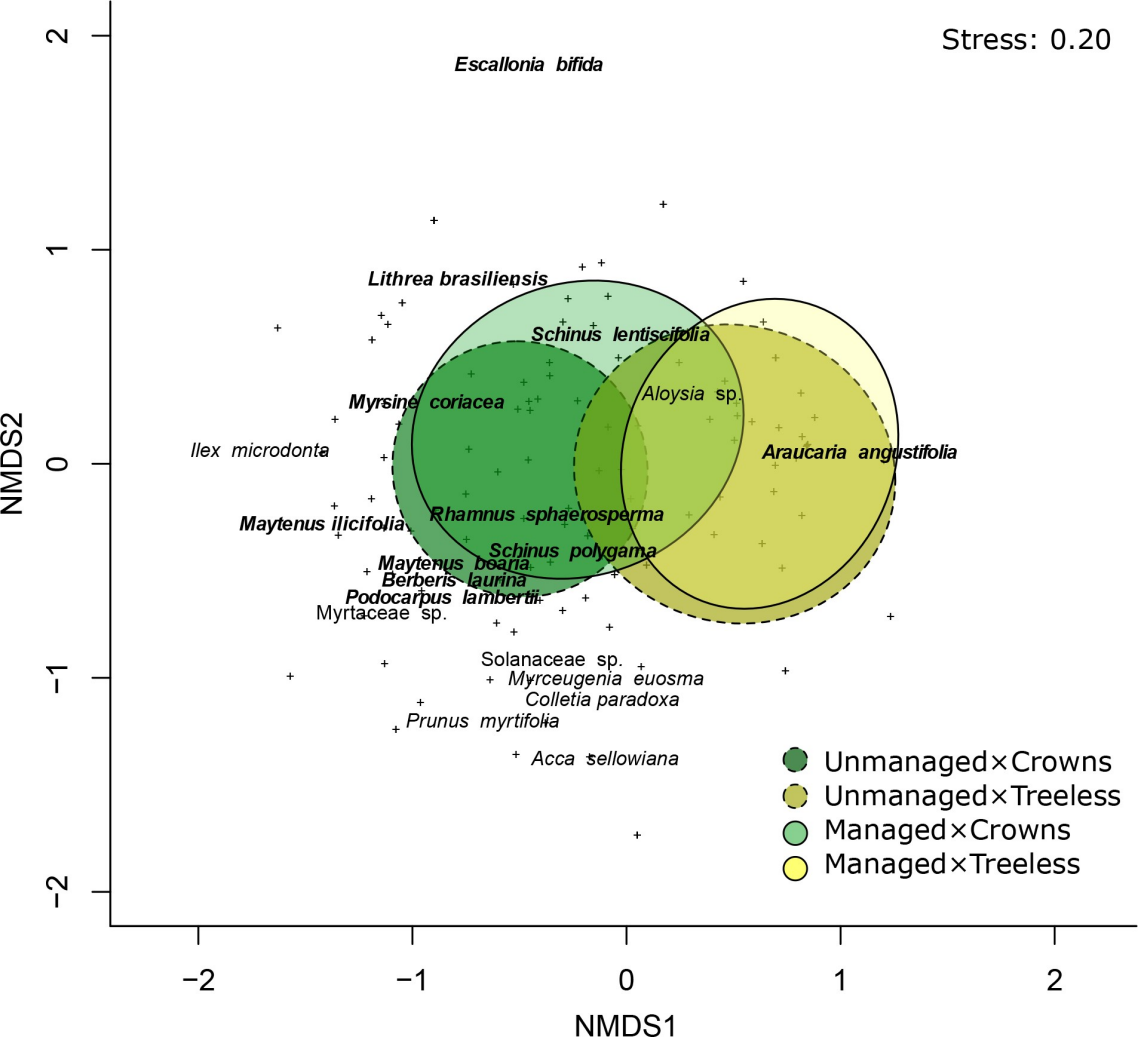
Non-metric multidimensional scaling (nMDS) ordination of saplings in relation to land management and araucaria crown influence in an upper-montane Araucaria forest, southern Brazil. Land management (unmanaged = dashed lines; managed = solid lines) and araucaria crown influence (beneath crowns = green tones; treeless areas = yellow tones). “+” represents sites and species in bold were significantly related to management and tree crown in the multivariate analysis. These ordinations are for visualization only; all statistical tests on the effects of land management and araucaria crown influence on community composition were conducted using multivariate response GLMs and taking the paired-sampling design into account.

Univariate results for species showed that type of land management and araucaria crown influence were correlated with 11 sapling species (58% of all species–S3 Table). There were fewer araucaria saplings beneath crowns than in treeless areas in both unmanaged and managed conditions (Fig 3 and S3 Table).

## Discussion

According to our results, the main consequence of the interactive effect between land management and crown influence was that land management overrode the facilitative effect provided by the nurse tree species. That is, although species richness was not generally affected by crown influence and land management, sapling abundance was normally higher in unmanaged conditions than managed conditions and increased beneath crowns for both management types. In addition, species composition was strongly affected by the interactive effect, differing in all combinations between land management and araucaria crown influence. Rock cover and grass volume affected richness and abundance of saplings in specific circumstances, while no effect of shrubs was found. Remarkably, araucaria saplings were found mostly in treeless areas in both managed and unmanaged conditions, indicating a possible preference for open spaces created by disturbances [55]. Although several studies had been carried out demonstrating the elements involved in patch formation and forest expansion in southern Brazilian highlands (*e*.*g*. [37], [19], [16], [36], [56]), as far as we know, our study is the first one to date that combines both land management and facilitation.

Araucaria forests have been shaped by human societies for millennia, transforming landscapes into biocultural systems [21].Therefore, it is essential to look at human activities and the relation of such activities with ecosystem structure [7]. Our results indicate that land management aiming to keep native grasslands hinders the establishment of most species but benefits araucaria seedlings and saplings. Therefore, local smallholders may play an important role in both araucaria protection and grassland maintenance. Local smallholders may also contribute to seed dispersal by voluntarily or involuntarily planting, since araucaria seeds represent an important source of food and income [21] and are frequently collected and transported from one place to another. As fire and cattle grazing are common management practices, they further contribute to grassland maintenance and diversity, hindering forest expansion [45,57]. Yet, most saplings were found beneath crowns in both managed and unmanaged conditions. The perch effect of araucaria trees [37,38] and the amelioration of environmental conditions beneath crowns [16] favor the establishment of woody species in these areas, which later grow into forest patches [37], when management is absent.

Grass volume and shrub cover are much higher in unmanaged conditions due to the lack of cattle grazing and fire. In southern Brazil, grasslands are dominated by tussock grasses and shrubs when cattle grazing is of low intensity or missing [32,58]. Over time, this vegetation can shift to shrublands and forests [45,57]. However, under such circumstances, high accumulation of flammable biomass increases the risk of catastrophic fires that can strongly reduce biodiversity [57] and negatively affect human wellbeing and cultural landscapes. In treeless areas, which had fewer species and individuals than beneath crowns, increasing grass volume further negatively affects sapling richness and abundance. Grasses and saplings may compete for resources, such as soil nutrients, water and light [35,36], and growth in height of grasses may be advantageous in competing for light, but may represent a disadvantage when herbivore pressure is high [59]. We believe that grasses may succeed over seedlings of woody species and saplings because of the physical barrier provided by their own fast growing aboveground biomass, which may hinder the establishment of most woody species, such as araucaria [36]. There was no effect of grass volume on sapling richness and abundance beneath crowns, possibly because of both facilitation and perch effects that araucaria trees exert on woody species and the interference of shade produced by araucaria crowns on grasses, resulting in lower grass volume.

The influence of abiotic factors, such as non-living objects (also called nurse objects, *e*.*g*., tree stumps and rocks), are still poorly explored [60]. In southern Brazilian grasslands, it has been shown that rocky outcrops favor the establishment of woody plants, contributing to patch formation and forest expansion [19]. Our results indicate that rocks may influence sapling abundance only under specific conditions. We found a very weak effect or no effect at all of rocks in treeless areas in managed and unmanaged conditions and beneath crowns in unmanaged areas. We believe that treeless areas in managed conditions are too exposed to harsh climate conditions, cattle herbivory and trampling and management fires, hindering the role of rocks in benefitting woody plants. Conversely, rocks positively affected sapling abundance beneath crowns in managed conditions. In this case, the combined positive effects of araucaria trees and rocks may have been responsible for this pattern. Rocks can improve plant germination, establishment and fitness by ameliorating conditions (such as shade, accumulation of soil and water), reducing grazing, protecting against mechanical and fire damage [10,17,19], and by never competing with its nurse [60]. Furthermore, rocks can also act as perches for frugivorous birds, refuge for small rodents and as a place for countermarking and a latrine for medium-sized omnivores [19].

In this study, the highest richness and abundance of saplings and the differences in community composition found beneath crowns in relation to treeless areas and to managed conditions further corroborate the role of araucaria trees in favoring forest species [37]. Conversely, low richness and abundance of saplings in managed conditions further reinforces the role of local smallholders in grassland maintenance. Although sapling richness decreases when cattle and fire are present, richness of grasses may increase when these components are appropriately managed [58]. Moreover, because araucaria saplings were found mostly in treeless areas in both unmanaged and managed conditions, it seems disturbances create gaps that benefit *A*. *angustifolia* [55].

Among other woody species, *Araucaria angustifolia* itself seemed to be negatively affected by its own crown in both managed and unmanaged conditions. Thus, land management allowing fire and cattle grazing seems to be neutral or even favorable for araucaria trees, at least when the fire interval is long enough to allow seedling development. Disturbances that create gaps, such as storms and fires, have been suggested as beneficial for *A*. *angustifolia*, at least within small spatial extents where seed rain is not disrupted [55,61]. Our results also indicate the role gaps created or maintained by fire have, together with additional management actions, on persistence and abundance of araucaria trees. Remarkably, similar land management actions were formerly made by pre-Columbian societies who managed forests and used fire [31]. These actions are now carried out by local smallholders [21], who contribute to niche construction [8] and promote the maintenance of a higher landscape diversity than expected without management.

Despite the small extent of the study site and geographical proximity of the locations, our sampling design allowed for reducing the effect of confounding factors, such as climate, soil type and depth, and topography. Moreover, both locations have a similar use history, since the land management employed in both areas in the past was the same. We also encourage future researches to include the relationships of humans with the landscape, since several species and their interactions can be affected by landscape domestication. To further understand the effects of land management along with ecological interactions, we recommend future studies to quantify land management, for example, by measuring grazing pressure and fire intensity. Understanding such relationships will allow for a better comprehension of ecologically relevant patterns and processes.

## Conclusions

Our study supports both the role araucaria trees have in promoting forest expansion and the importance of local smallholders in maintaining grasslands. On the one hand, araucaria trees contribute to increasing sapling species richness and abundance, and in changing community composition beneath their crowns. On the other hand, land management actions taken by local smallholders help in maintaining natural grasslands by hindering forest expansion through fire and cattle grazing. We demonstrate the key roles these two major components (land management actions and araucaria trees) have in plant community assemblage, contributing to landscape diversity maintenance in upper-montane regions. Disturbances promoted by land management can favor the maintenance of threatened landscapes and trees, such as highland grasslands and araucaria trees. Our results also clarify patterns and processes that may emerge in natural highland grasslands, such as the conversion of grasslands into forests and modification of cultural landscapes when the most significant management actions (grazing and fire) are excluded. Consequently, maximal diversity can be achieved by a balanced set of both protected areas and maintenance of traditional management practices.

## Supporting information

S1 Table Summary of main variables measured under distinct land management and araucaria crown influence and covariates (grass volume, rock and shrub cover) in an upper-montane Araucaria forest, southern Brazil. Except for sampling area, richness and abundance, other variables were mean ± standard deviation.
(PDF)

S2 Table Complete GLMMs for evaluating species richness and abundance of saplings in relation to land management, araucaria crown influence and covariates (grass volume, rock and shrub cover) in an upper-montane Araucaria forest, southern Brazil. Significant P-values are in bold. Unman = unmanaged sites; Man = managed sites; Crowns = beneath araucaria crowns; Treeless = treeless areas; Grass = grass volume; Rock = rock cover.
(PDF)

S3 Table Univariate results of multivariate tests. This table shows how species abundances were affected by the interaction between land management and araucaria crown influence as well as the direction of the effect. Overall Test Stats = overall effect for each species. LR = likelihood ratio test, Unman = Unmanaged conditions, Man = Managed conditions, Canopies = beneath crowns, Treeless = Treeless areas. Significant effects are in bold. “+” indicate positive effects and “-” indicate negative effects (from the second level compared to the first).
(PDF)

S1 Fig Differences among measured variables for unmanaged and managed locations in an upper-montane Araucaria forest, southern Brazil. A = terrain inclination (°); B = rock cover (m^2^); C = elevation (m); D = Circumference at Breast Height (CBH) of sampled araucaria trees (cm). Circles represent samples (average of the sampled pair–except for CBH).
(PDF)

S1 File Data set used for analyses. (PDF)
