# Supplementary material for: Interaction of land management and araucaria trees in the maintenance of landscape diversity in the highlands of southern Brazil
Source: PLoS One. 2018 Nov 21;13(11):e0206805. doi: 10.1371/journal.pone.0206805 (PMC6248941; doi:10.1371/journal.pone.0206805)
Supplement: S1 Table — Except for sampling area, richness and abundance, other variables were mean ± standard deviation. (PDF) [file pone.0206805.s001.pdf]

Araucaria crown influence	Variables	Managed	Unmanaged	Total
**Beneath crowns**	Sampling area (m^2)	1065.3	2809.1	3874.4
	Grass volume (m^3)	0.013±0.009	0.021±0.016	0.019±0.015
	Shrub cover (m^2)	0.0018±0.006	0.064±0.061	0.046±0.059
	Rock cover (m^2)	0.025±0.036	0.031±0.042	0.029±0.041
	Total richness	10	15	15
	Total abundance	186	884	1070
**Treeless areas**	Sampling area (m^2)	1065.3	2809.1	3874.4
	Grass volume (m^3)	0.013±0.013	0.036±0.018	0.029±0.020
	Shrub cover (m^2)	0.0009±0.003	0.052±0.051	0.037±0.049
	Rock cover (m^2)	0.030±0.037	0.014±0.025	0.019±0.030
	Total richness	10	12	14
	Total abundance	92	138	230
**Total**	Sampling area (m^2)	2130.6	5618.2	7748.8
	Grass volume (m^3)	0.013±0.011	0.028±0.019	0.024±0.018
	Shrub cover (m^2)	0.001±0.004	0.058±0.019	0.041±0.054
	Rock cover (m^2)	0.028 ±0.037	0.022±0.036	0.024±0.036
	Total richness	13	17	19
	Total abundance	278	1022	1300
