# Supplementary material for: Interaction of land management and araucaria trees in the maintenance of landscape diversity in the highlands of southern Brazil
Source: PLoS One. 2018 Nov 21;13(11):e0206805. doi: 10.1371/journal.pone.0206805 (PMC6248941; doi:10.1371/journal.pone.0206805)
Supplement: S2 Table — Significant P-values are in bold. Unman = unmanaged sites; Man = managed sites; Crowns = beneath araucaria crowns; Treeless = treeless areas; Grass = grass volume; Rock = rock cover. (PDF) [file pone.0206805.s002.pdf]

Model	Model ID	Coefficients	Estimate	Std. Error	z	P
Sapling species richness	ric.7	(Intercept)	-3.245	0.274	-11.86	**< 0.0001**
		Treeless	0.185	0.311	0.59	0.5530
		Unman	1.098	0.310	3.54	**0.0004**
		Unman×Treeless	-0.768	0.421	-1.83	**0.0679**
		Man×Crowns×Grass	19.232	17.654	1.09	0.2760
		Man×Treeless×Grass	-19.123	18.625	-1.03	0.3045
		Unman×Crowns×Grass	-6.646	6.156	-1.08	0.2803
		Unman×Treeless×Grass	-19.908	7.799	-2.55	**0.0107**
Sapling abundance	abu.2	(Intercept)	-3.428	0.412	-8.33	**< 0.0001**
		Treeless	0.962	0.483	1.99	**0.0463**
		Unman	2.393	0.497	4.81	**<0.0001**
		Unman×Treeless	-2.085	0.616	-3.39	**0.0007**
		Man×Crowns×Rock	18.079	5.244	3.45	**0.0006**
		Man×Treeless×Rock	5.035	5.576	0.9	0.3666
		Unman×Crowns×Rock	2.175	3.295	0.66	0.5091
		Unman×Treeless×Rock	-6.171	6.735	-0.92	0.3595
		Man×Crowns×Grass	34.061	22.142	1.54	0.1240
		Man×Treeless×Grass	-41.692	23.562	-1.77	0.0768
		Unman×Crowns×Grass	-13.800	9.314	-1.48	0.1384
		Unman×Treeless×Grass	-19.026	8.839	-2.15	**0.0314**
