# Supplementary material for: Interaction of land management and araucaria trees in the maintenance of landscape diversity in the highlands of southern Brazil
Source: PLoS One. 2018 Nov 21;13(11):e0206805. doi: 10.1371/journal.pone.0206805 (PMC6248941; doi:10.1371/journal.pone.0206805)
Supplement: S3 Table — This table shows how species abundances were affected by the interaction between land management and araucaria crown influence as well as the direction of the effect. Overall Test Stats = overall effect for each species. LR = likelihood ratio test, Unman = Unmanaged conditions, Man = Managed conditions, Canopies = beneath crowns, Treeless = Treeless areas. Significant effects are in bold. “+” indicate positive effects and “-” indicate negative effects (from the second level compared to the first). (PDF) [file pone.0206805.s003.pdf]

	Overall Test Stats		Unman×Crowns x Man×Crowns		Unman×Crowns x Unman×Treeless		Unman×Crowns x Man×Treeless		Unman×Treeless x Man×Treeless		Unman×Treeless x Man×Crowns		Man×Crowns x Man×Treeless	
**Species**	**LR**	**P**	**LR**	**P**	**LR**	**P**	**LR**	**P**	**LR**	**P**	**LR**	**P**	**LR**	**P**
Acca sellowiana (O.Berg) Burret	2.301	0.4147	1.265	0.6429	0.777	0.5756	0.157	0.8854	0.295	0.9763	0.157	0.8854	0.004	0.9376
Aloysia sp.	1.570	0.4147	0.887	0.6429	0.140	0.8572	0.000	0.9488	0.589	0.9201	0.000	0.9488	0.000	1.0000
Araucaria angustifolia (Bertol.) Kuntze	17.828	**0.0023**	1.397	0.6429	8.432$^{(+)}$	**0.0035**	1.346	0.5938	0.118	0.9827	1.346	0.5938	9.867$^{(+)}$	**0.0086**
Berberis laurina Billb.	31.748	**0.0001**	3.298	0.3288	28.599$^{(-)}$	**0.0001**	4.720	0.0764	1.683	0.8067	4.720	0.0764	1.630	0.5355
Colletia paradoxa (Spreng.) Escal.	3.870	0.2063	0.000	0.6429	0.000	0.9901	0.001	0.8858	2.471	0.6041	0.001	0.8858	1.430	0.5355
Escallonia bifida Link & Otto	11.727	**0.0144**	8.002$^{(+)}$	**0.0330**	0.000	0.8572	0.000	0.9488	2.409	0.6201	0.000	0.9488	1.850	0.5355
Ilex microdonta Reissek	2.105	0.4147	0.719	0.6429	1.386	0.2702	0.001	0.8858	0.000	0.9998	0.001	0.8858	0.000	0.9376
Lithrea brasiliensis Marchand	57.180	**0.0001**	13.686$^{(-)}$	**0.0037**	43.806$^{(-)}$	**0.0001**	7.252$^{(+)}$	**0.0173**	0.013	0.9827	7.252$^{(-)}$	**0.0173**	1.644	0.5355
Maytenus boaria Molina	47.019	**0.0001**	10.491$^{(-)}$	**0.0123**	42.887$^{(-)}$	**0.0001**	16.687$^{(+)}$	**0.0003**	8.125$^{(+)}$	**0.0431**	16.687$^{(-)}$	**0.0003**	0.058	0.9376
Maytenus ilicifolia Mart. ex Reissek	11.784	**0.0141**	7.561$^{(+)}$	**0.0384**	0.000	0.9901	0.002	0.8858	0.000	0.9998	0.002	0.8858	4.383	0.1127
Myrceugenia euosma (O.Berg) D.Legrand	3.780	0.2143	0.000	0.8712	0.000	0.9901	0.001	0.8858	2.394	0.6240	0.001	0.8858	1.386	0.5355
Myrsine coriacea (Sw.) R.Br. ex Roem. & Schult.	52.587	**0.0001**	8.356$^{(-)}$	**0.0330**	36.549$^{(-)}$	0.0001	0.033	0.8858	1.527	0.8067	0.033	0.8858	9.248$^{(-)}$	**0.0104**
Myrtaceae sp.	6.712	0.0861	3.144	0.3288	3.017	0.0559	0.000	0.9488	0.632	0.9108	0.000	0.9488	0.000	1.0000
Podocarpus lambertii Klotzsch ex Endl.	9.982	**0.0264**	1.508	0.6429	7.579$^{(-)}$	**0.0035**	0.000	0.9488	0.000	0.9827	0.000	0.9488	1.085	0.5355
Prunus myrtifolia (L.) Urb.	4.211	0.1812	1.439	0.6429	2.772	0.0559	0.001	0.8858	0.000	0.9827	0.001	0.8858	0.000	1.0000
Rhamnus sphaerosperma Sw.	14.796	**0.0055**	1.681	0.6429	11.443$^{(-)}$	**0.0008**	0.000	0.9488	0.000	0.9998	0.000	0.9488	2.098	0.4708
Schinus lentiscifolia Marchand	15.432	**0.0055**	3.597	0.3149	11.407$^{(-)}$	**0.0008**	1.108	0.5938	0.061	0.9827	1.108	0.5938	0.694	0.6440
Schinus polygama (Cav.) Cabrera	21.543	**0.0006**	5.953	0.1010	14.055$^{(-)}$	**0.0003**	0.088	0.8854	0.712	0.8910	0.088	0.8854	1.312	0.5355
Solanaceae sp.	2.105	0.4147	0.719	0.6429	1.386	0.2702	0.001	0.8858	0.000	0.9998	0.001	0.8858	0.000	1.0000
