# Supplementary figures and images for: Interaction of land management and araucaria trees in the maintenance of landscape diversity in the highlands of southern Brazil
Source: PLoS One. 2018 Nov 21;13(11):e0206805. doi: 10.1371/journal.pone.0206805 (PMC6248941; doi:10.1371/journal.pone.0206805)

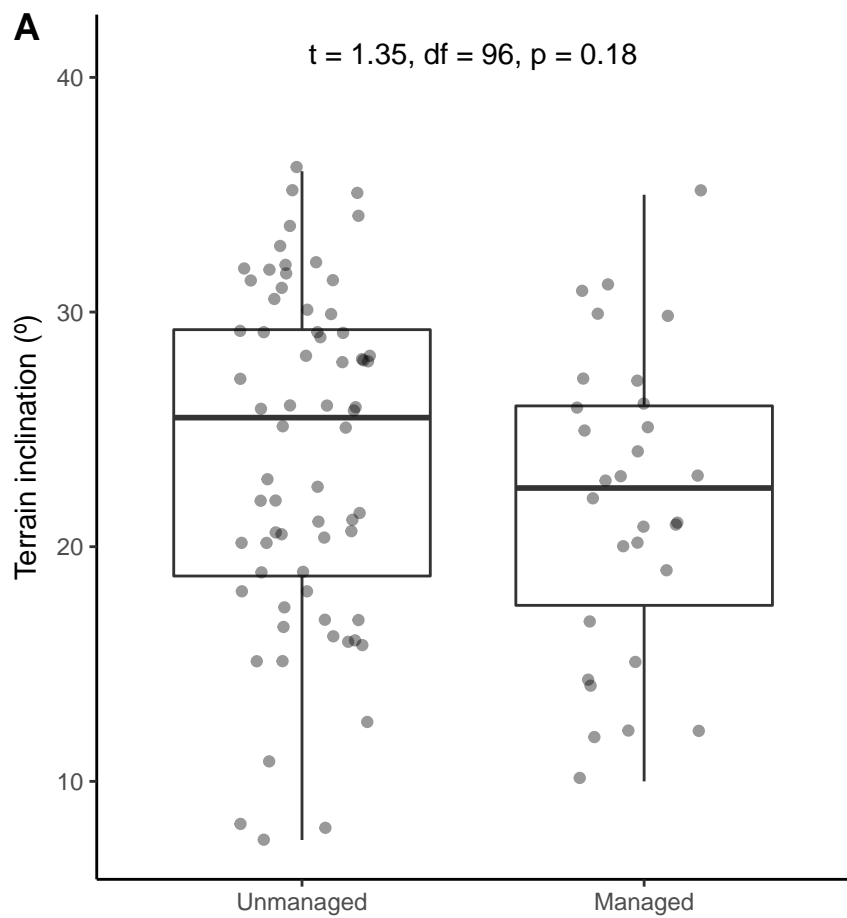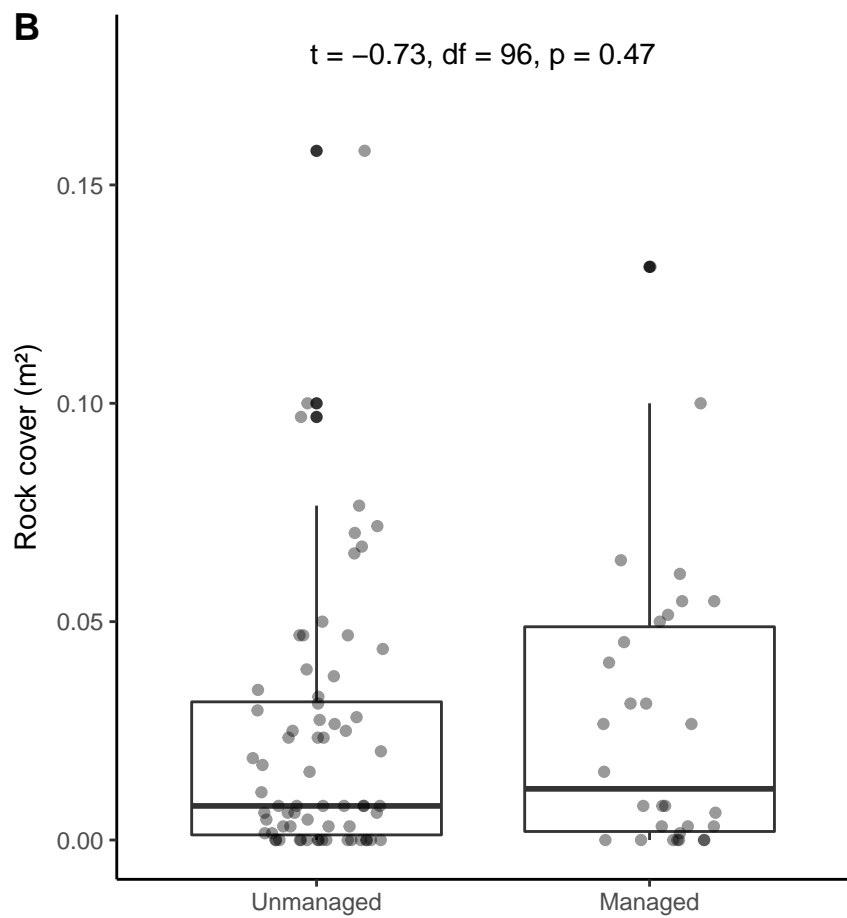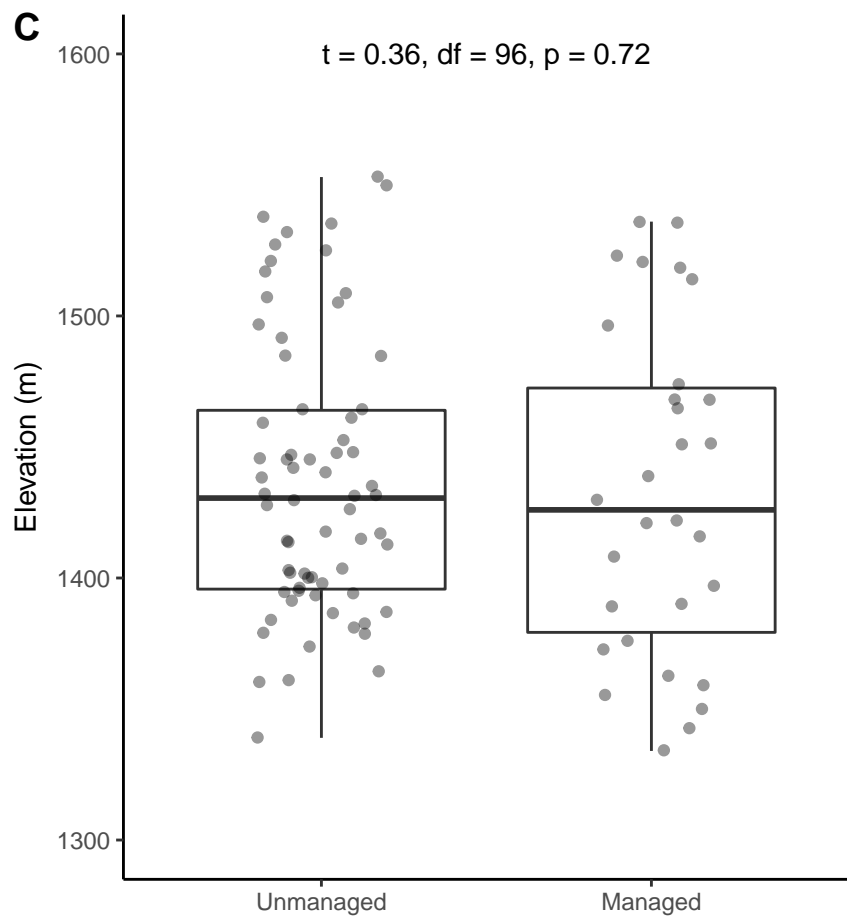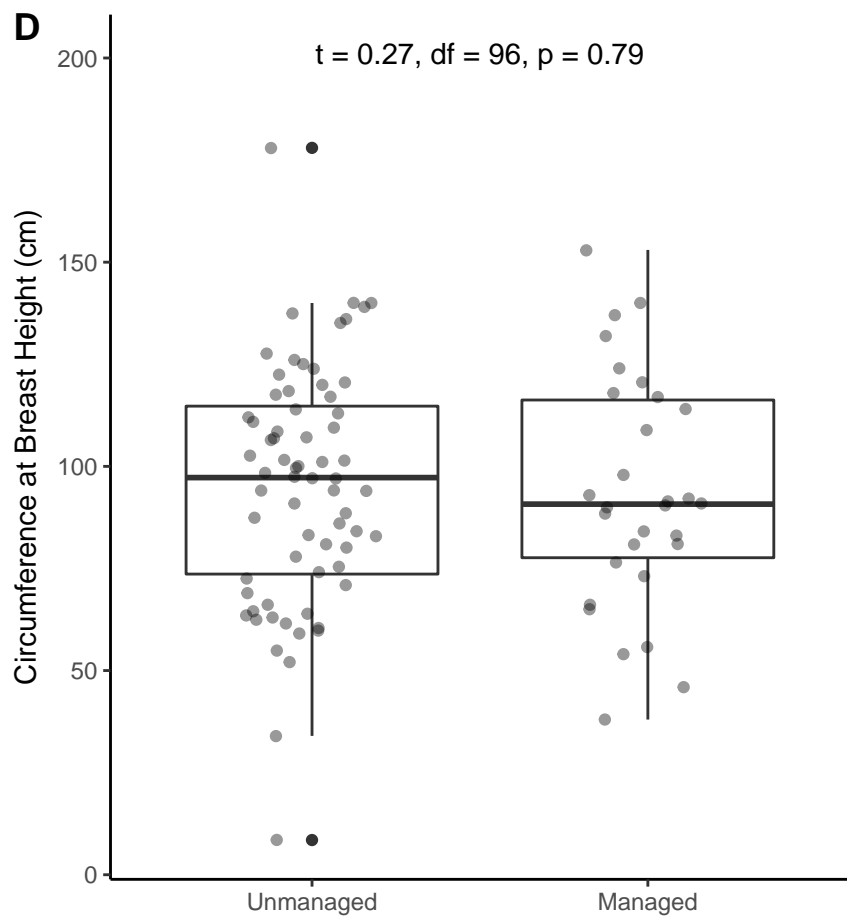

Supplement: S1 Fig — A = terrain inclination (°); B = rock cover (m2); C = elevation (m); D = Circumference at Breast Height (CBH) of sampled araucaria trees (cm). Circles represent samples (average of the sampled pair–except for CBH). (PDF) [file pone.0206805.s004.pdf]
