# Supplementary material for: Interaction of land management and araucaria trees in the maintenance of landscape diversity in the highlands of southern Brazil
Source: PLoS One. 2018 Nov 21;13(11):e0206805. doi: 10.1371/journal.pone.0206805 (PMC6248941; doi:10.1371/journal.pone.0206805)
Supplement: S1 File — (PDF) [file pone.0206805.s005.pdf]

Sample	Location	crown.influence	block	sampling.area	land.management	Rock.cover	Shrub.cover	Grass.volume	abundance	richness	Acca_sellowiana	Aloysia_sp	Araucaria_angustifolia	Berberis_laurina	Colletia_paradoxa
A1	L1	crown	1	38.485	unmanaged	0.1000	0.1094	0.01341	25	6	0	0	0	12	0
B1	L1	treeless	1	38.485	unmanaged	0.0344	0.0000	0.01954	0	0	0	0	0	0	0
A2	L1	crown	2	19.635	unmanaged	0.0125	0.0000	0.02452	23	6	0	0	0	4	0
B2	L1	treeless	2	19.635	unmanaged	0.0281	0.0625	0.01395	3	2	0	0	0	0	0
A3	L1	crown	3	38.485	unmanaged	0.0550	0.0031	0.02218	8	3	0	0	1	0	0
B3	L1	treeless	3	38.485	unmanaged	0.0000	0.0000	0.03458	0	0	0	0	0	0	0
A4	L1	crown	4	78.540	unmanaged	0.0125	0.1094	0.01846	9	3	0	0	0	2	0
B4	L1	treeless	4	78.540	unmanaged	0.0000	0.0188	0.02198	0	0	0	0	0	0	0
A5	L1	crown	5	50.265	unmanaged	0.0469	0.0000	0.00023	63	5	2	0	0	0	0
B5	L1	treeless	5	50.265	unmanaged	0.0000	0.0313	0.01200	17	3	0	0	0	3	0
A6	L1	crown	6	11.045	unmanaged	0.0000	0.0875	0.03588	16	3	0	0	0	0	0
B6	L1	treeless	6	11.045	unmanaged	0.0063	0.1094	0.02572	0	0	0	0	0	0	0
A7	L1	crown	7	28.274	unmanaged	0.0094	0.0000	0.01178	13	4	1	0	0	7	0
B7	L1	treeless	7	28.274	unmanaged	0.0000	0.1063	0.02078	4	3	2	0	0	1	0
A8	L1	crown	8	19.635	unmanaged	0.0000	0.1125	0.02487	10	3	0	0	0	6	0
B8	L1	treeless	8	19.635	unmanaged	0.0156	0.0000	0.03223	2	2	0	0	1	0	0
A9	L1	crown	9	28.274	unmanaged	0.0313	0.0938	0.01229	5	2	0	0	0	1	0
B9	L1	treeless	9	28.274	unmanaged	0.0000	0.0000	0.02109	2	2	0	0	0	0	0
A10	L1	crown	10	19.635	unmanaged	0.0313	0.0469	0.02748	7	3	0	0	0	2	0
B10	L1	treeless	10	19.635	unmanaged	0.0156	0.1063	0.02183	2	2	0	0	1	0	0
A11	L1	crown	11	44.179	unmanaged	0.0000	0.0625	0.00947	25	8	2	0	1	4	0
B11	L1	treeless	11	44.179	unmanaged	0.0000	0.1375	0.04406	2	2	0	0	1	0	0
A12	L1	crown	12	23.758	unmanaged	0.0000	0.1063	0.03127	12	6	0	0	1	1	0
B12	L1	treeless	12	23.758	unmanaged	0.0031	0.0969	0.03965	7	3	0	0	3	0	0
A13	L1	crown	13	50.265	unmanaged	0.0000	0.1125	0.01969	20	7	3	0	0	4	0
B13	L1	treeless	13	50.265	unmanaged	0.0031	0.0000	0.02345	6	3	0	0	1	0	0
A14	L1	crown	14	50.265	unmanaged	0.0000	0.0969	0.03765	15	7	2	0	1	1	0
B14	L1	treeless	14	50.265	unmanaged	0.0000	0.0000	0.06297	7	1	0	0	7	0	0
A15	L1	crown	15	78.540	unmanaged	0.1438	0.0313	0.00264	25	7	1	0	1	5	0
B15	L1	treeless	15	78.540	unmanaged	0.0000	0.0250	0.06125	0	0	0	0	0	0	0

A16	L1	crown	16	7.069	unmanaged	0.0313	0.0000	0.02523	4	4	0	0	0	0	0
B16	L1	treeless	16	7.069	unmanaged	0.0250	0.0375	0.03319	0	0	0	0	0	0	0
A17	L1	crown	17	38.485	unmanaged	0.0000	0.0031	0.03870	5	3	0	0	0	3	0
B17	L1	treeless	17	38.485	unmanaged	0.0000	0.0844	0.05438	0	0	0	0	0	0	0
A18	L1	crown	18	28.274	unmanaged	0.0000	0.1031	0.03517	1	1	0	0	0	0	0
B18	L1	treeless	18	28.274	unmanaged	0.0469	0.0219	0.02713	0	0	0	0	0	0	0
A19	L1	crown	19	33.183	unmanaged	0.0000	0.0625	0.03440	3	2	0	0	1	0	0
B19	L1	treeless	19	33.183	unmanaged	0.0156	0.0000	0.03340	1	1	0	0	1	0	0
A20	L1	crown	20	66.476	unmanaged	0.0750	0.0000	0.00477	40	9	0	0	2	17	0
B20	L1	treeless	20	66.476	unmanaged	0.0000	0.2500	0.00619	11	4	4	0	0	5	0
A21	L1	crown	21	19.635	unmanaged	0.0125	0.0000	0.00305	27	6	0	0	0	9	0
B21	L1	treeless	21	19.635	unmanaged	0.0000	0.0000	0.02093	6	2	0	0	0	2	0
A22	L1	crown	22	24.630	unmanaged	0.0594	0.0844	0.00651	7	3	0	0	2	2	0
B22	L1	treeless	22	24.630	unmanaged	0.0188	0.0000	0.02168	5	2	0	0	4	1	0
A23	L1	crown	23	33.183	unmanaged	0.0500	0.0156	0.00846	27	4	0	0	1	4	0
B23	L1	treeless	23	33.183	unmanaged	0.0125	0.0000	0.02538	2	1	0	0	2	0	0
A24	L1	crown	24	20.428	unmanaged	0.0656	0.0281	0.01268	1	1	0	0	0	1	0
B24	L1	treeless	24	20.428	unmanaged	0.0281	0.0438	0.01858	0	0	0	0	0	0	0
A25	L1	crown	25	15.904	unmanaged	0.0906	0.0000	0.00545	13	3	0	0	0	0	0
B25	L1	treeless	25	15.904	unmanaged	0.0094	0.1094	0.02208	0	0	0	0	0	0	0
A26	L1	crown	26	28.274	unmanaged	0.0938	0.0875	0.01753	13	4	0	0	0	9	0
B26	L1	treeless	26	28.274	unmanaged	0.0000	0.1188	0.04765	1	1	0	0	0	0	0
A27	L1	crown	27	32.170	unmanaged	0.0063	0.0625	0.03124	7	4	0	0	0	2	0
B27	L1	treeless	27	32.170	unmanaged	0.0000	0.0844	0.02172	2	2	0	0	1	0	0
A28	L1	crown	28	30.191	unmanaged	0.0094	0.0000	0.04765	23	5	0	0	1	0	0
B28	L1	treeless	28	30.191	unmanaged	0.0000	0.0063	0.03000	0	0	0	0	0	0	0
A29	L1	crown	29	50.265	unmanaged	0.0156	0.0094	0.02112	3	2	0	0	0	0	0
B29	L1	treeless	29	50.265	unmanaged	0.0000	0.0063	0.02844	2	1	0	0	2	0	0
A31	L1	crown	31	7.069	unmanaged	0.0375	0.1813	0.00837	22	5	0	0	0	5	0
B31	L1	treeless	31	7.069	unmanaged	0.0156	0.0000	0.03135	1	1	0	0	0	0	0
A32	L1	crown	32	52.810	unmanaged	0.0000	0.0000	0.01627	16	7	1	0	1	2	0
B32	L1	treeless	32	52.810	unmanaged	0.0063	0.0000	0.05001	4	2	0	0	2	2	0
A33	L1	crown	33	50.265	unmanaged	0.0000	0.0313	0.04447	3	2	0	0	0	0	0
B33	L1	treeless	33	50.265	unmanaged	0.0000	0.0313	0.04125	8	2	0	0	7	0	0

A34	L1	crown	34	50.265	unmanaged	0.0000	0.0375	0.02213	8	6	0	0	1	1	0
B34	L1	treeless	34	50.265	unmanaged	0.0000	0.1000	0.02344	4	1	0	0	4	0	0
A35	L1	crown	35	40.715	unmanaged	0.0656	0.0000	0.00802	3	2	0	0	0	0	0
B35	L1	treeless	35	40.715	unmanaged	0.0000	0.0313	0.03209	4	1	0	0	4	0	0
A36	L1	crown	36	50.265	unmanaged	0.0000	0.0625	0.04035	11	4	0	0	1	0	0
B36	L1	treeless	36	50.265	unmanaged	0.0156	0.0313	0.03604	3	1	0	0	3	0	0
A37	L1	crown	37	78.540	unmanaged	0.0000	0.1938	0.00974	6	3	0	0	2	0	0
B37	L1	treeless	37	78.540	unmanaged	0.0000	0.1156	0.02969	1	1	1	0	0	0	0
A38	L1	crown	38	50.265	unmanaged	0.0000	0.2500	0.00891	15	4	0	0	0	0	0
B38	L1	treeless	38	50.265	unmanaged	0.0000	0.1094	0.02578	7	2	0	0	6	0	0
A39	L1	crown	39	10.179	unmanaged	0.0188	0.0000	0.03530	1	1	0	0	1	0	0
B39	L1	treeless	39	10.179	unmanaged	0.0313	0.1063	0.04484	0	0	0	0	0	0	0
A40	L1	crown	40	9.079	unmanaged	0.0844	0.0000	0.01967	2	2	0	0	0	1	0
B40	L1	treeless	40	9.079	unmanaged	0.0688	0.0625	0.02198	0	0	0	0	0	0	0
A41	L1	crown	41	12.566	unmanaged	0.1000	0.0000	0.01322	1	1	0	0	0	0	0
B41	L1	treeless	41	12.566	unmanaged	0.0938	0.0313	0.01396	0	0	0	0	0	0	0
A42	L1	crown	42	12.566	unmanaged	0.0000	0.0000	0.08641	0	0	0	0	0	0	0
B42	L1	treeless	42	12.566	unmanaged	0.0000	0.0000	0.10547	0	0	0	0	0	0	0
A43	L1	crown	43	50.265	unmanaged	0.0000	0.1188	0.05304	1	1	0	0	0	1	0
B43	L1	treeless	43	50.265	unmanaged	0.0219	0.0313	0.02857	0	0	0	0	0	0	0
A45	L1	crown	45	69.398	unmanaged	0.1406	0.0500	0.00586	3	2	0	0	0	0	0
B45	L1	treeless	45	69.398	unmanaged	0.0000	0.0813	0.05609	0	0	0	0	0	0	0
A46	L1	crown	46	8.042	unmanaged	0.1219	0.0500	0.01249	4	3	0	0	1	0	0
B46	L1	treeless	46	8.042	unmanaged	0.0781	0.0875	0.03169	1	1	1	0	0	0	0
A47	L1	crown	47	28.274	unmanaged	0.1906	0.1250	0.00148	7	3	0	0	0	1	0
B47	L1	treeless	47	28.274	unmanaged	0.1250	0.0719	0.01633	2	2	0	0	1	0	0
A48	L1	crown	48	10.179	unmanaged	0.0000	0.0781	0.06359	0	0	0	0	0	0	0
B48	L1	treeless	48	10.179	unmanaged	0.0000	0.0906	0.05797	0	0	0	0	0	0	0
A49	L1	crown	49	43.008	unmanaged	0.0000	0.0938	0.00790	3	2	0	0	0	2	0
B49	L1	treeless	49	43.008	unmanaged	0.0156	0.0688	0.06182	1	1	0	0	1	0	0
A50	L1	crown	50	47.784	unmanaged	0.0000	0.1563	0.02016	6	4	0	0	2	1	0
B50	L1	treeless	50	47.784	unmanaged	0.0000	0.0813	0.04859	1	1	0	0	1	0	0
A51	L1	crown	51	63.617	unmanaged	0.0000	0.0781	0.03141	52	7	0	0	3	10	0
B51	L1	treeless	51	63.617	unmanaged	0.0000	0.0313	0.07391	0	0	0	0	0	0	0

A52	L1	crown	52	23.758	unmanaged	0.0000	0.0438	0.03656	3	1	0	0	3	0	0
B52	L1	treeless	52	23.758	unmanaged	0.0125	0.0594	0.04008	1	1	0	0	1	0	0
A53	L1	crown	53	38.485	unmanaged	0.0531	0.0313	0.01740	15	6	0	0	2	2	0
B53	L1	treeless	53	38.485	unmanaged	0.0344	0.0000	0.02453	3	1	0	0	3	0	0
A54	L1	crown	54	66.476	unmanaged	0.0344	0.0063	0.01650	13	3	0	0	0	0	0
B54	L1	treeless	54	66.476	unmanaged	0.0000	0.0000	0.03125	3	2	0	0	2	0	0
A55	L1	crown	55	12.566	unmanaged	0.0000	0.1125	0.04413	12	5	2	0	0	1	0
B55	L1	treeless	55	12.566	unmanaged	0.0000	0.0500	0.05688	3	2	2	0	1	0	0
A56	L1	crown	56	28.274	unmanaged	0.0000	0.1563	0.02209	2	2	0	0	0	0	0
B56	L1	treeless	56	28.274	unmanaged	0.0000	0.0813	0.04188	4	1	0	0	4	0	0
A57	L1	crown	57	38.485	unmanaged	0.0156	0.1125	0.01359	0	0	0	0	0	0	0
B57	L1	treeless	57	38.485	unmanaged	0.0000	0.1094	0.05891	1	1	0	0	1	0	0
A58	L1	crown	58	24.630	unmanaged	0.0688	0.1938	0.00657	9	5	1	0	1	0	0
B58	L1	treeless	58	24.630	unmanaged	0.0000	0.0188	0.03595	1	1	0	0	0	0	0
A59	L1	crown	59	21.237	unmanaged	0.0000	0.1594	0.04106	3	1	0	0	0	0	0
B59	L1	treeless	59	21.237	unmanaged	0.0469	0.1406	0.03161	1	1	0	0	1	0	0
A60	L1	crown	60	24.630	unmanaged	0.0000	0.0688	0.01723	2	1	0	0	0	0	0
B60	L1	treeless	60	24.630	unmanaged	0.0156	0.0313	0.02857	1	1	0	0	1	0	0
A61	L1	crown	61	45.365	unmanaged	0.0000	0.0750	0.02297	2	1	0	0	0	0	0
B61	L1	treeless	61	45.365	unmanaged	0.0000	0.1531	0.03489	0	0	0	0	0	0	0
A63	L1	crown	63	58.088	unmanaged	0.0000	0.0000	0.00088	14	7	3	0	0	3	0
B63	L1	treeless	63	58.088	unmanaged	0.0000	0.0625	0.03472	3	2	2	0	1	0	0
A64	L1	crown	64	30.191	unmanaged	0.0125	0.1969	0.01730	5	4	0	0	0	1	0
B64	L1	treeless	64	30.191	unmanaged	0.0000	0.0156	0.07141	0	0	0	0	0	0	0
A65	L1	crown	65	28.274	unmanaged	0.0156	0.0625	0.01547	3	1	0	0	0	0	0
B65	L1	treeless	65	28.274	unmanaged	0.0219	0.0000	0.05090	6	3	0	0	3	0	0
A66	L1	crown	66	33.183	unmanaged	0.0594	0.0625	0.00876	21	5	1	0	0	5	0
B66	L1	treeless	66	33.183	unmanaged	0.0719	0.0000	0.02331	2	1	0	0	0	0	0
A67	L1	crown	67	44.179	unmanaged	0.0500	0.0625	0.01350	9	3	0	0	0	2	0
B67	L1	treeless	67	44.179	unmanaged	0.0000	0.0000	0.04984	0	0	0	0	0	0	0
A68	L1	crown	68	38.485	unmanaged	0.0938	0.0000	0.00166	15	7	2	0	0	2	0
B68	L1	treeless	68	38.485	unmanaged	0.0000	0.0000	0.01597	3	3	1	0	0	1	0
A69	L1	crown	69	22.902	unmanaged	0.0594	0.0000	0.02089	5	2	0	0	0	1	0
B69	L1	treeless	69	22.902	unmanaged	0.0000	0.0156	0.07500	0	0	0	0	0	0	0

A70	L1	crown	70	33.183	unmanaged	0.0063	0.0000	0.01519	3	3	0	0	0	1	0
B70	L1	treeless	70	33.183	unmanaged	0.0000	0.0625	0.01989	2	2	0	0	1	1	0
A71	L1	crown	71	19.635	unmanaged	0.0000	0.0000	0.01392	16	7	0	2	1	3	0
B71	L1	treeless	71	19.635	unmanaged	0.0000	0.0000	0.05359	5	3	0	1	1	0	0
A72	L1	crown	72	44.179	unmanaged	0.0000	0.0000	0.03184	44	9	1	0	3	3	0
B72	L1	treeless	72	44.179	unmanaged	0.0000	0.0313	0.01855	11	5	1	0	1	1	0
A73	L1	crown	73	32.170	unmanaged	0.0000	0.0625	0.00012	16	4	3	0	0	1	0
B73	L1	treeless	73	32.170	unmanaged	0.0000	0.0000	0.03016	3	3	1	0	0	1	0
A74	L2	crown	74	28.274	managed	0.0500	0.0000	0.03713	25	7	0	0	0	3	0
B74	L2	treeless	74	28.274	managed	0.0594	0.0000	0.02335	0	0	0	0	0	0	0
A75	L2	crown	75	50.265	managed	0.0000	0.0031	0.00367	7	2	4	0	0	3	0
B75	L2	treeless	75	50.265	managed	0.0000	0.0000	0.00414	4	1	4	0	0	0	0
A76	L2	crown	76	50.265	managed	0.1531	0.0000	0.00254	22	4	0	0	0	15	0
B76	L2	treeless	76	50.265	managed	0.1094	0.0000	0.00105	10	4	1	0	0	7	0
A77	L2	crown	77	15.904	managed	0.0531	0.0000	0.00584	13	4	0	0	0	4	0
B77	L2	treeless	77	15.904	managed	0.0000	0.0000	0.06719	1	1	0	0	0	0	0
A78	L2	crown	78	50.265	managed	0.0906	0.0000	0.00777	20	6	0	0	1	1	0
B78	L2	treeless	78	50.265	managed	0.1094	0.0063	0.00659	11	4	0	0	1	1	0
A79	L2	crown	79	3.142	managed	0.0313	0.0000	0.03254	1	1	0	0	0	0	0
B79	L2	treeless	79	3.142	managed	0.0219	0.0000	0.02410	0	0	0	0	0	0	0
A80	L2	crown	80	24.630	managed	0.0000	0.0094	0.02422	6	3	0	0	0	0	0
B80	L2	treeless	80	24.630	managed	0.0625	0.0031	0.00820	9	3	0	0	0	5	0
A81	L2	crown	81	63.617	managed	0.0000	0.0000	0.01219	8	5	0	0	0	0	0
B81	L2	treeless	81	63.617	managed	0.1000	0.0000	0.00248	6	3	0	0	4	0	0
A82	L2	crown	82	19.635	managed	0.0000	0.0000	0.02641	0	0	0	0	0	0	0
B82	L2	treeless	82	19.635	managed	0.0000	0.0000	0.03781	0	0	0	0	0	0	0
A83	L2	crown	83	7.069	managed	0.0031	0.0000	0.01466	0	0	0	0	0	0	0
B83	L2	treeless	83	7.069	managed	0.0031	0.0000	0.01281	0	0	0	0	0	0	0
A84	L2	crown	84	12.566	managed	0.0000	0.0000	0.01703	0	0	0	0	0	0	0
B84	L2	treeless	84	12.566	managed	0.0000	0.0000	0.02094	0	0	0	0	0	0	0
A85	L2	crown	85	63.617	managed	0.0000	0.0000	0.02438	0	0	0	0	0	0	0
B85	L2	treeless	85	63.617	managed	0.0313	0.0000	0.01818	0	0	0	0	0	0	0
A86	L2	crown	86	19.635	managed	0.0406	0.0000	0.01544	0	0	0	0	0	0	0
B86	L2	treeless	86	19.635	managed	0.0500	0.0000	0.01288	1	1	0	0	1	0	0

A87	L2	crown	87	24.630	managed	0.0469	0.0000	0.01917	0	0	0	0	0	0	0
B87	L2	treeless	87	24.630	managed	0.0344	0.0000	0.01765	2	2	0	0	1	0	0
A88	L2	crown	88	34.212	managed	0.0000	0.0000	0.02266	0	0	0	0	0	0	0
B88	L2	treeless	88	34.212	managed	0.0000	0.0000	0.01875	1	1	0	0	1	0	0
A89	L2	crown	89	45.365	managed	0.0000	0.0000	0.00737	0	0	0	0	0	0	0
B89	L2	treeless	89	45.365	managed	0.0000	0.0000	0.00750	0	0	0	0	0	0	0
A90	L2	crown	90	58.088	managed	0.0000	0.0000	0.01063	0	0	0	0	0	0	0
B90	L2	treeless	90	58.088	managed	0.0156	0.0000	0.01011	2	1	0	0	2	0	0
A91	L2	crown	91	45.365	managed	0.0156	0.0000	0.01054	1	1	0	0	1	0	0
B91	L2	treeless	91	45.365	managed	0.0000	0.0000	0.01703	1	1	0	0	1	0	0
A92	L2	crown	92	40.715	managed	0.0000	0.0031	0.00960	3	3	0	0	1	0	0
B92	L2	treeless	92	40.715	managed	0.0000	0.0031	0.00313	3	2	0	0	2	0	0
A93	L2	crown	93	21.237	managed	0.0063	0.0000	0.00746	0	0	0	0	0	0	0
B93	L2	treeless	93	21.237	managed	0.0000	0.0000	0.01000	1	1	0	0	1	0	0
A94	L2	crown	94	63.617	managed	0.0625	0.0000	0.00438	6	3	0	0	2	0	0
B94	L2	treeless	94	63.617	managed	0.0594	0.0000	0.00286	5	3	0	0	2	0	0
A95	L2	crown	95	28.274	managed	0.0844	0.0031	0.00259	4	3	1	0	2	0	0
B95	L2	treeless	95	28.274	managed	0.0438	0.0000	0.01173	0	0	0	0	0	0	0
A96	L2	crown	96	36.317	managed	0.0188	0.0000	0.01200	0	0	0	0	0	0	0
B96	L2	treeless	96	36.317	managed	0.0844	0.0000	0.00217	3	1	0	0	3	0	0
A97	L2	crown	97	50.265	managed	0.0219	0.0000	0.00884	3	3	0	0	0	0	0
B97	L2	treeless	97	50.265	managed	0.0875	0.0000	0.00345	4	2	0	0	3	0	0
A98	L2	crown	98	34.212	managed	0.0156	0.0000	0.00882	4	3	0	0	0	1	0
B98	L2	treeless	98	34.212	managed	0.0000	0.0000	0.00594	2	2	0	0	1	0	0
A99	L2	crown	99	7.069	managed	0.0031	0.0313	0.00725	0	0	0	0	0	0	0
B99	L2	treeless	99	7.069	managed	0.0094	0.0156	0.00526	4	1	0	0	4	0	0
A100	L2	crown	100	19.635	managed	0.0000	0.0063	0.00762	3	2	0	0	1	0	0
B100	L2	treeless	100	19.635	managed	0.0031	0.0000	0.01096	1	1	0	0	1	0	0
A101	L2	crown	101	36.317	managed	0.0563	0.0000	0.00488	1	1	0	0	0	0	0
B101	L2	treeless	101	36.317	managed	0.0063	0.0000	0.00868	2	2	0	0	1	0	0
A102	L2	crown	102	60.821	managed	0.0063	0.0000	0.00312	3	3	0	0	1	0	0
B102	L2	treeless	102	60.821	managed	0.0000	0.0000	0.01000	14	8	0	0	1	2	3
A103	L2	crown	103	50.265	managed	0.0000	0.0000	0.00842	8	4	0	0	0	4	0
B103	L2	treeless	103	50.265	managed	0.0000	0.0000	0.00820	5	2	0	0	4	0	0

Escalloni a_bifida	Ilex_micr odonta	Lithraea_ brasiliens is	Maytenu s_boaria	Maytenu s_muelle ri	Myrceug enia_euo sma	Myrsine_ coriacea	Myrtacea e_sp	Podocarp us_lamb ertii	Prunus_ myrtifoli a	Rhamnus _sphaero sperma	Schinus_l entiscifol ius	Schinus_ polygam us	Solanace ae_sp
0	0	8	1	0	0	0	0	2	0	0	1	1	0
0	0	0	0	0	0	0	0	0	0	0	0	0	0
0	0	10	2	0	0	0	0	5	0	0	1	1	0
0	0	2	0	0	0	0	0	0	0	0	1	0	0
0	0	6	0	0	0	0	0	0	0	0	1	0	0
0	0	0	0	0	0	0	0	0	0	0	0	0	0
0	0	5	0	0	0	0	2	0	0	0	0	0	0
0	0	0	0	0	0	0	0	0	0	0	0	0	0
0	0	50	1	0	0	0	0	0	0	0	9	1	0
0	0	8	0	0	0	0	0	0	0	0	6	0	0
0	0	14	0	0	0	1	0	0	0	0	1	0	0
0	0	0	0	0	0	0	0	0	0	0	0	0	0
0	0	1	0	0	0	0	0	0	0	0	4	0	0
0	0	0	0	0	0	0	0	0	0	0	1	0	0
0	0	2	0	0	0	0	0	0	0	0	2	0	0
0	0	0	0	0	0	0	0	0	0	0	1	0	0
0	0	0	0	0	0	0	0	0	0	0	4	0	0
0	0	1	0	0	0	0	0	0	0	0	1	0	0
0	0	3	0	0	0	0	0	0	0	0	2	0	0
0	0	0	0	0	0	0	0	0	0	0	1	0	0
0	0	9	1	0	0	4	0	0	0	0	3	1	0
0	0	0	0	0	0	0	0	0	0	0	1	0	0
0	0	3	1	0	0	3	0	0	0	0	3	0	0
0	0	3	0	0	0	0	0	0	0	0	1	0	0
0	0	5	3	0	0	1	0	0	0	1	3	0	0
0	0	2	0	0	0	0	0	0	0	0	3	0	0
0	0	3	0	0	0	4	0	0	0	0	2	2	0
0	0	0	0	0	0	0	0	0	0	0	0	0	0
0	0	2	1	0	0	14	0	0	0	0	0	0	1
0	0	0	0	0	0	0	0	0	0	0	0	0	0

0	0	1	1	0	0	0	0	0	0	0	1	1	0
0	0	0	0	0	0	0	0	0	0	0	0	0	0
0	0	1	1	0	0	0	0	0	0	0	0	0	0
0	0	0	0	0	0	0	0	0	0	0	0	0	0
0	0	0	1	0	0	0	0	0	0	0	0	0	0
0	0	0	0	0	0	0	0	0	0	0	0	0	0
0	0	0	0	0	0	2	0	0	0	0	0	0	0
0	0	0	0	0	0	0	0	0	0	0	0	0	0
0	0	1	5	0	0	9	2	0	1	1	0	2	0
0	0	0	1	0	0	0	1	0	0	0	0	0	0
0	0	2	2	0	0	9	2	0	0	0	0	3	0
0	0	0	0	0	0	4	0	0	0	0	0	0	0
0	0	0	0	0	0	3	0	0	0	0	0	0	0
0	0	0	0	0	0	0	0	0	0	0	0	0	0
0	0	0	1	0	0	21	0	0	0	0	0	0	0
0	0	0	0	0	0	0	0	0	0	0	0	0	0
0	0	0	0	0	0	0	0	0	0	0	0	0	0
0	0	0	0	0	0	0	0	0	0	0	0	0	0
0	0	1	2	0	0	10	0	0	0	0	0	0	0
0	0	0	0	0	0	0	0	0	0	0	0	0	0
0	0	1	0	0	0	2	0	0	0	0	1	0	0
0	0	0	0	0	0	0	0	0	0	0	1	0	0
0	0	1	0	0	0	1	0	0	0	0	3	0	0
0	0	0	0	0	0	0	0	0	0	0	0	1	0
0	0	8	7	0	0	2	0	0	0	0	5	0	0
0	0	0	0	0	0	0	0	0	0	0	0	0	0
0	0	2	0	0	0	1	0	0	0	0	0	0	0
0	0	0	0	0	0	0	0	0	0	0	0	0	0
0	0	13	0	0	0	1	0	0	0	0	1	2	0
0	0	1	0	0	0	0	0	0	0	0	0	0	0
0	0	7	0	0	0	1	0	1	0	0	3	0	0
0	0	0	0	0	0	0	0	0	0	0	0	0	0
0	0	2	0	0	0	1	0	0	0	0	0	0	0
0	0	1	0	0	0	0	0	0	0	0	0	0	0

0	0	3	0	0	0	1	0	0	0	1	0	1	0
0	0	0	0	0	0	0	0	0	0	0	0	0	0
0	0	1	0	0	0	2	0	0	0	0	0	0	0
0	0	0	0	0	0	0	0	0	0	0	0	0	0
0	0	8	1	0	0	1	0	0	0	0	0	0	0
0	0	0	0	0	0	0	0	0	0	0	0	0	0
0	0	2	2	0	0	0	0	0	0	0	0	0	0
0	0	0	0	0	0	0	0	0	0	0	0	0	0
0	1	6	4	0	0	4	0	0	0	0	0	0	0
0	0	1	0	0	0	0	0	0	0	0	0	0	0
0	0	0	0	0	0	0	0	0	0	0	0	0	0
0	0	0	0	0	0	0	0	0	0	0	0	0	0
0	0	0	1	0	0	0	0	0	0	0	0	0	0
0	0	0	0	0	0	0	0	0	0	0	0	0	0
0	0	1	0	0	0	0	0	0	0	0	0	0	0
0	0	0	0	0	0	0	0	0	0	0	0	0	0
0	0	0	0	0	0	0	0	0	0	0	0	0	0
0	0	0	0	0	0	0	0	0	0	0	0	0	0
0	0	0	0	0	0	0	0	0	0	0	0	0	0
0	0	0	0	0	0	0	0	0	0	0	0	0	0
0	0	1	0	0	0	2	0	0	0	0	0	0	0
0	0	0	0	0	0	0	0	0	0	0	0	0	0
0	0	2	0	0	0	1	0	0	0	0	0	0	0
0	0	0	0	0	0	0	0	0	0	0	0	0	0
0	0	0	3	0	0	3	0	0	0	0	0	0	0
0	0	0	0	0	0	1	0	0	0	0	0	0	0
0	0	0	0	0	0	0	0	0	0	0	0	0	0
0	0	0	0	0	0	0	0	0	0	0	0	0	0
0	0	1	0	0	0	0	0	0	0	0	0	0	0
0	0	0	0	0	0	0	0	0	0	0	0	0	0
0	0	1	2	0	0	0	0	0	0	0	0	0	0
0	0	0	0	0	0	0	0	0	0	0	0	0	0
0	0	5	2	0	0	26	0	1	0	5	0	0	0
0	0	0	0	0	0	0	0	0	0	0	0	0	0

0	0	0	0	0	0	0	0	0	0	0	0	0	0
0	0	0	0	0	0	0	0	0	0	0	0	0	0
0	0	5	1	0	0	4	0	0	0	1	0	0	0
0	0	0	0	0	0	0	0	0	0	0	0	0	0
0	0	8	2	0	0	3	0	0	0	0	0	0	0
0	0	1	0	0	0	0	0	0	0	0	0	0	0
0	0	3	1	0	0	5	0	0	0	0	0	0	0
0	0	0	0	0	0	0	0	0	0	0	0	0	0
0	0	1	0	0	0	1	0	0	0	0	0	0	0
0	0	0	0	0	0	0	0	0	0	0	0	0	0
0	0	0	0	0	0	0	0	0	0	0	0	0	0
0	0	0	0	0	0	0	0	0	0	0	0	0	0
0	0	5	1	0	0	1	0	0	0	0	0	0	0
0	0	0	0	0	0	1	0	0	0	0	0	0	0
0	0	3	0	0	0	0	0	0	0	0	0	0	0
0	0	0	0	0	0	0	0	0	0	0	0	0	0
0	0	2	0	0	0	0	0	0	0	0	0	0	0
0	0	0	0	0	0	0	0	0	0	0	0	0	0
0	0	2	0	0	0	0	0	0	0	0	0	0	0
0	0	0	0	0	0	0	0	0	0	0	0	0	0
0	0	4	1	0	0	0	0	1	1	0	0	1	0
0	0	0	0	0	0	0	0	0	0	0	0	0	0
0	0	2	0	0	0	1	0	0	0	0	1	0	0
0	0	0	0	0	0	0	0	0	0	0	0	0	0
0	0	3	0	0	0	0	0	0	0	0	0	0	0
0	0	2	0	0	0	0	0	0	0	0	1	0	0
0	0	10	0	0	0	1	0	0	0	0	4	0	0
0	0	2	0	0	0	0	0	0	0	0	0	0	0
0	0	1	0	0	0	0	0	0	0	0	6	0	0
0	0	0	0	0	0	0	0	0	0	0	0	0	0
0	0	6	0	0	0	1	0	0	0	1	2	1	0
0	0	0	0	0	0	0	0	0	0	0	1	0	0
0	0	4	0	0	0	0	0	0	0	0	0	0	0
0	0	0	0	0	0	0	0	0	0	0	0	0	0

0	0	1	0	0	0	0	0	0	0	0	1	0	0
0	0	0	0	0	0	0	0	0	0	0	0	0	0
0	0	6	1	0	0	1	0	0	0	2	0	0	0
0	0	3	0	0	0	0	0	0	0	0	0	0	0
0	0	15	3	0	0	13	1	0	0	3	0	2	0
0	0	7	0	0	0	1	0	0	0	0	0	0	0
0	0	11	0	0	0	1	0	0	0	0	0	0	0
0	0	1	0	0	0	0	0	0	0	0	0	0	0
7	0	3	1	4	0	6	0	0	0	0	1	0	0
0	0	0	0	0	0	0	0	0	0	0	0	0	0
0	0	0	0	0	0	0	0	0	0	0	0	0	0
0	0	0	0	0	0	0	0	0	0	0	0	0	0
0	0	3	0	3	0	1	0	0	0	0	0	0	0
0	0	1	0	0	0	0	0	0	0	0	1	0	0
0	0	5	0	3	0	1	0	0	0	0	0	0	0
1	0	0	0	0	0	0	0	0	0	0	0	0	0
12	0	3	1	0	0	0	0	0	0	0	2	0	0
0	0	7	2	0	0	0	0	0	0	0	0	0	0
0	0	1	0	0	0	0	0	0	0	0	0	0	0
0	0	0	0	0	0	0	0	0	0	0	0	0	0
1	0	2	0	0	0	3	0	0	0	0	0	0	0
0	0	3	1	0	0	0	0	0	0	0	0	0	0
0	0	3	1	0	0	1	0	0	0	2	1	0	0
0	0	0	1	0	0	0	0	0	0	0	1	0	0
0	0	0	0	0	0	0	0	0	0	0	0	0	0
0	0	0	0	0	0	0	0	0	0	0	0	0	0
0	0	0	0	0	0	0	0	0	0	0	0	0	0
0	0	0	0	0	0	0	0	0	0	0	0	0	0
0	0	0	0	0	0	0	0	0	0	0	0	0	0
0	0	0	0	0	0	0	0	0	0	0	0	0	0
0	0	0	0	0	0	0	0	0	0	0	0	0	0
0	0	0	0	0	0	0	0	0	0	0	0	0	0
0	0	0	0	0	0	0	0	0	0	0	0	0	0
0	0	0	0	0	0	0	0	0	0	0	0	0	0

0	0	0	0	0	0	0	0	0	0	0	0	0	0
0	0	1	0	0	0	0	0	0	0	0	0	0	0
0	0	0	0	0	0	0	0	0	0	0	0	0	0
0	0	0	0	0	0	0	0	0	0	0	0	0	0
0	0	0	0	0	0	0	0	0	0	0	0	0	0
0	0	0	0	0	0	0	0	0	0	0	0	0	0
0	0	0	0	0	0	0	0	0	0	0	0	0	0
0	0	0	0	0	0	0	0	0	0	0	0	0	0
0	0	0	0	0	0	0	0	0	0	0	0	0	0
0	0	0	0	0	0	0	0	0	0	0	0	0	0
0	0	1	0	0	0	0	0	0	0	0	1	0	0
0	0	0	0	0	0	0	0	0	0	0	1	0	0
0	0	0	0	0	0	0	0	0	0	0	0	0	0
0	0	0	0	0	0	0	0	0	0	0	0	0	0
0	0	3	0	0	0	0	0	0	0	0	1	0	0
0	0	2	0	0	0	0	0	0	0	0	1	0	0
0	0	0	0	0	0	0	0	0	0	0	1	0	0
0	0	0	0	0	0	0	0	0	0	0	0	0	0
0	0	0	0	0	0	0	0	0	0	0	0	0	0
0	0	0	0	0	0	0	0	0	0	0	0	0	0
0	0	1	0	0	0	1	0	0	0	0	1	0	0
0	0	0	0	0	0	0	0	0	0	0	1	0	0
0	0	2	0	0	0	0	0	0	0	0	1	0	0
0	0	0	0	0	0	0	0	0	0	0	1	0	0
0	0	0	0	0	0	0	0	0	0	0	0	0	0
0	0	0	0	0	0	0	0	0	0	0	0	0	0
0	0	0	0	0	0	0	0	0	0	0	2	0	0
0	0	0	0	0	0	0	0	0	0	0	0	0	0
0	0	0	0	0	0	0	0	0	0	0	1	0	0
0	0	0	0	0	0	0	0	0	0	0	1	0	0
0	0	1	0	0	0	1	0	0	0	0	0	0	0
0	0	3	2	0	1	1	0	0	0	0	1	0	0
0	0	0	2	0	0	0	0	1	0	0	0	1	0
0	0	1	0	0	0	0	0	0	0	0	0	0	0
